# Supplementary figures and images for: eIF4A, a target of siRNA derived from rice stripe virus, negatively regulates antiviral autophagy by interacting with ATG5 in Nicotiana benthamiana
Source: PLoS Pathog. 2021 Sep 29;17(9):e1009963. doi: 10.1371/journal.ppat.1009963 (PMC8504976; doi:10.1371/journal.ppat.1009963)

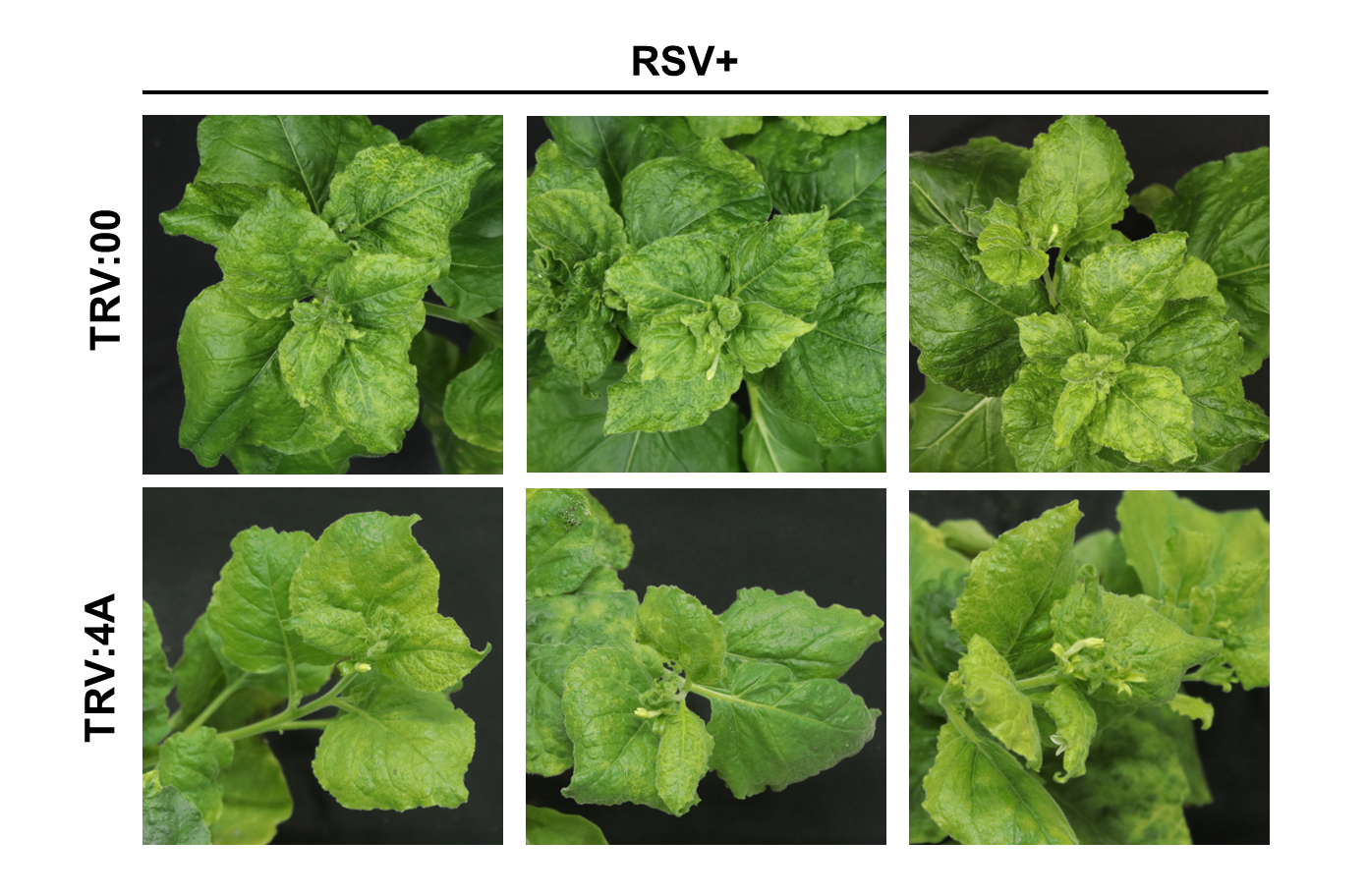

Supplement: S1 Fig — Typical RSV symptoms such as stunting and leaf-twisting with yellow mosaicism appeared on all TRV:00 control plants, while only 25% of RSV-inoculated NbeIF4A-silenced plants showed yellow mosaicism on leaves at 20 dpi, which was less pronounced than the control. Silencing of NbeIF4A caused leaf-twisting, which may interfere with the observation of RSV symptoms. The most obvious symptom of RSV infection is yellow and mosaic leaves, which was attenuated in NbeIF4A-silenced plants. (TIF) [file ppat.1009963.s001.TIF]

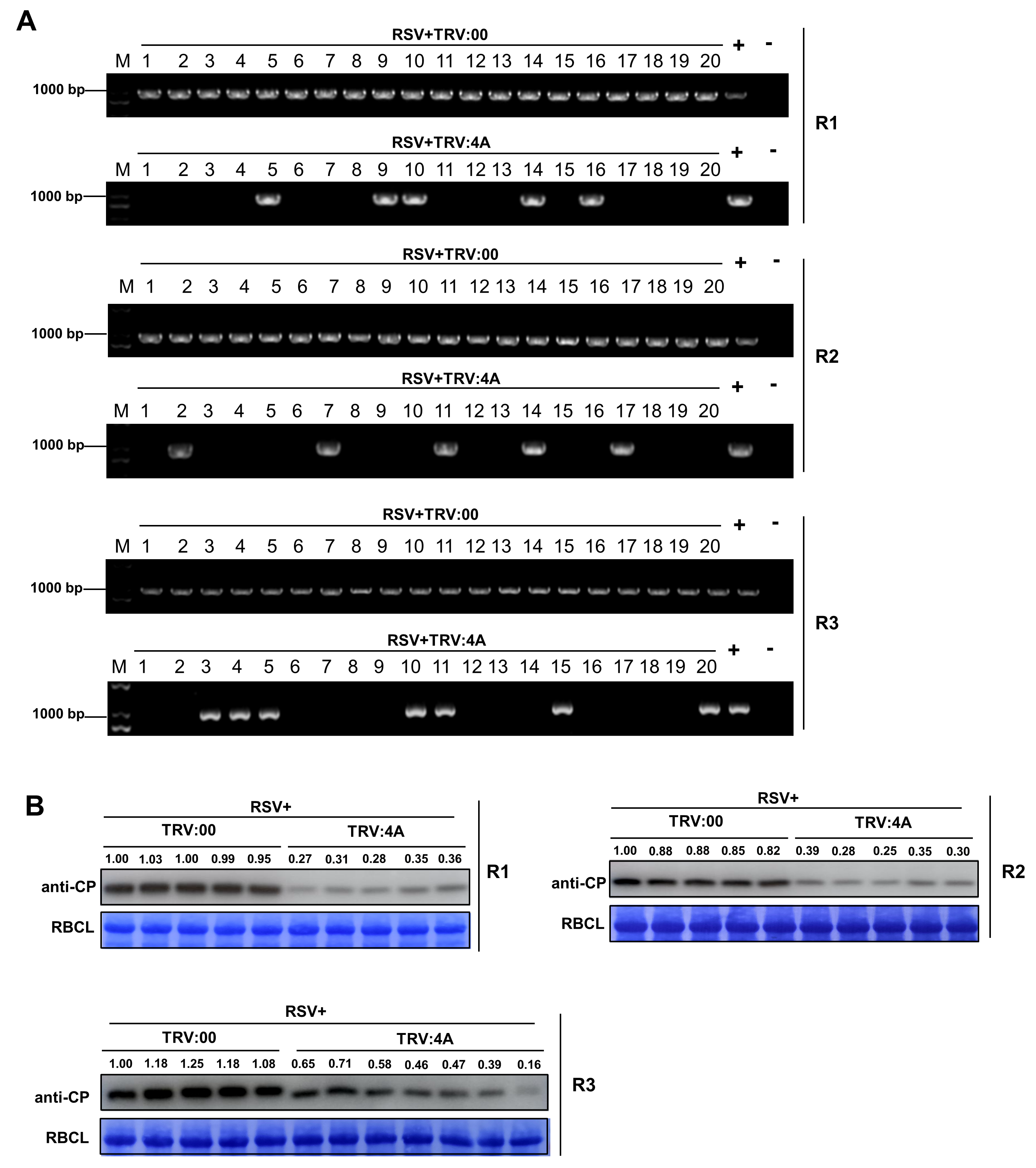

Supplement: S2 Fig — (A) RT-PCR to analyze systemic RSV infection. Results from three replicates (R1, R2 and R3) are shown. Twenty plants per replicate were subjected to infection by TRV:4A or TRV:00. (B) Immunoblot analysis of RSV CP accumulation in RSV-infected NbeIF4A-silenced plants in three replicates (R1, R2, and R3). RSV-infected TRV:00-infected plants were used as a control. Band intensity was analyzed by ImageJ. (TIF) [file ppat.1009963.s002.TIF]

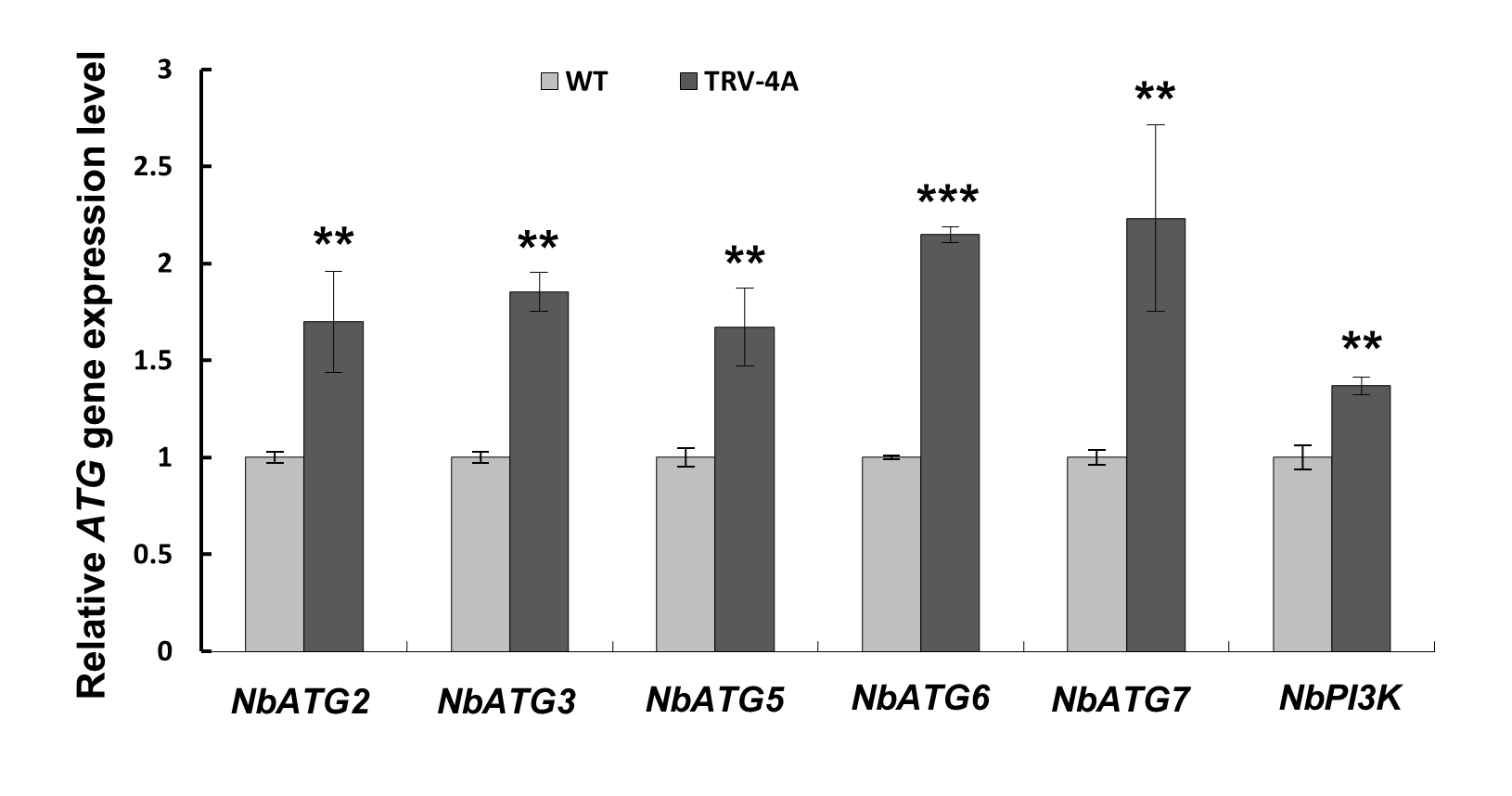

Supplement: S3 Fig — RT-qPCR was used to measure the relative transcript level of genes in the autophagy pathway. NbActin served as the internal control. Error bars indicate standard error from three individual experiments. Asterisks indicate significant differences by Student’s t-test compared to the control (*, p<0.05; **, p<0.01). (TIF) [file ppat.1009963.s003.TIF]

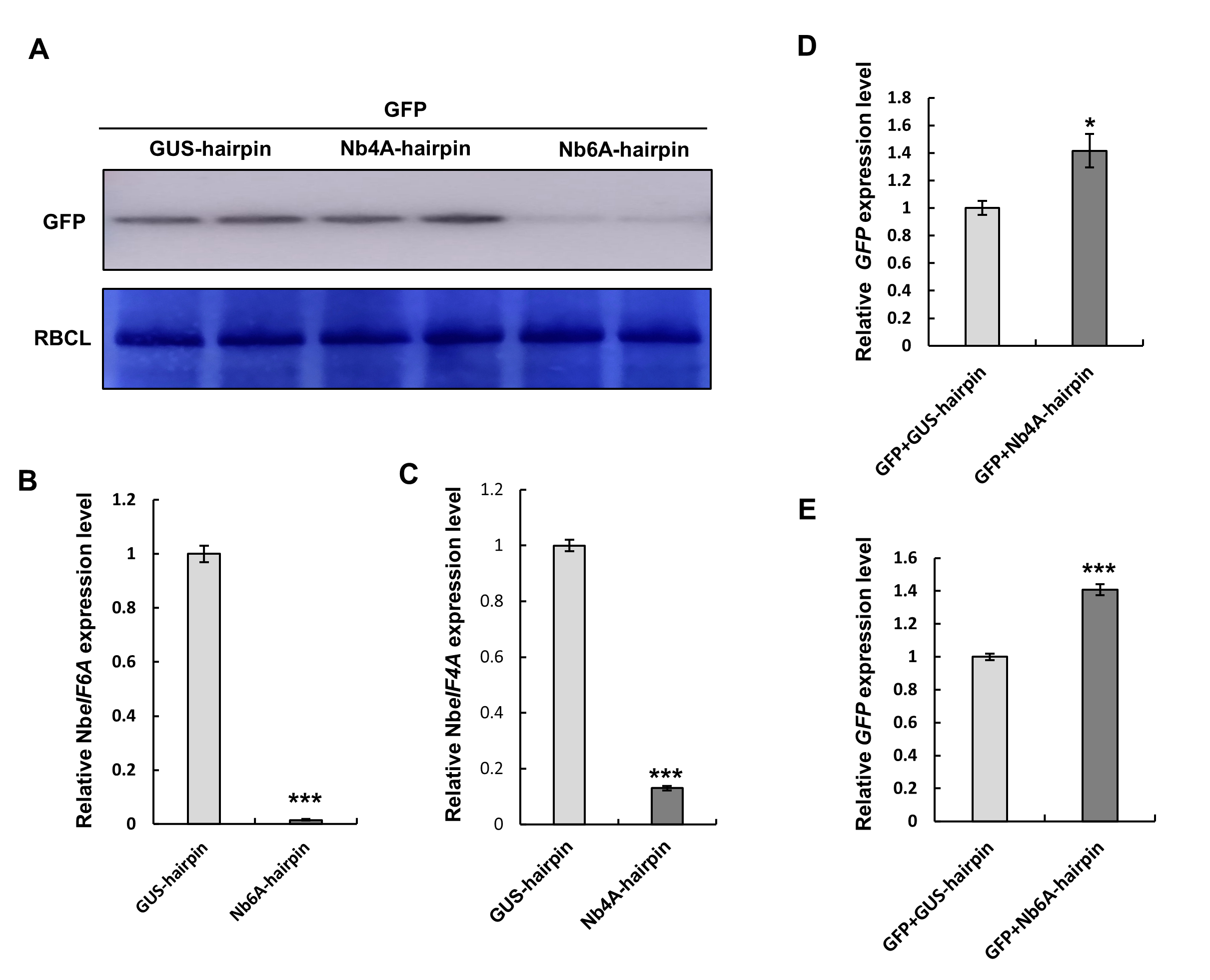

Supplement: S4 Fig — (A) Immunoblot analysis of GFP expressed in NbeIF4A-silenced cells (4A-hairpin), NbeIF6A-silenced cells (6A-hairpin), or non-silenced cells (GUS-hairpin). Rubisco large subunit was used as a loading control. Silencing of NbeIF6A was used as positive control, as it impaired protein translation. (B) RT-qPCR analysis of NbeIF6A expression in NbeIF6A-silenced cells. Asterisks indicate significant differences by Student’s t-test (***, p < 0.005) compared to the control. (C) RT-qPCR analysis of NbeIF4A mRNA levels in NbeIF4A-silenced cells. Asterisks indicate significant differences by Student’s t-test (***, p < 0.005) compared to the control. (D) RT-qPCR analysis of GFP mRNA levels in NbeIF6A-silenced cells. Asterisks indicate significant differences by Student’s t-test (*, p < 0.05) compared to the control. (E) RT-qPCR analysis of GFP mRNA levels in NbeIF4A-silenced cells. Asterisks indicate significant differences by Student’s t-test (***, p < 0.005) compared to the control. (TIF) [file ppat.1009963.s004.TIF]

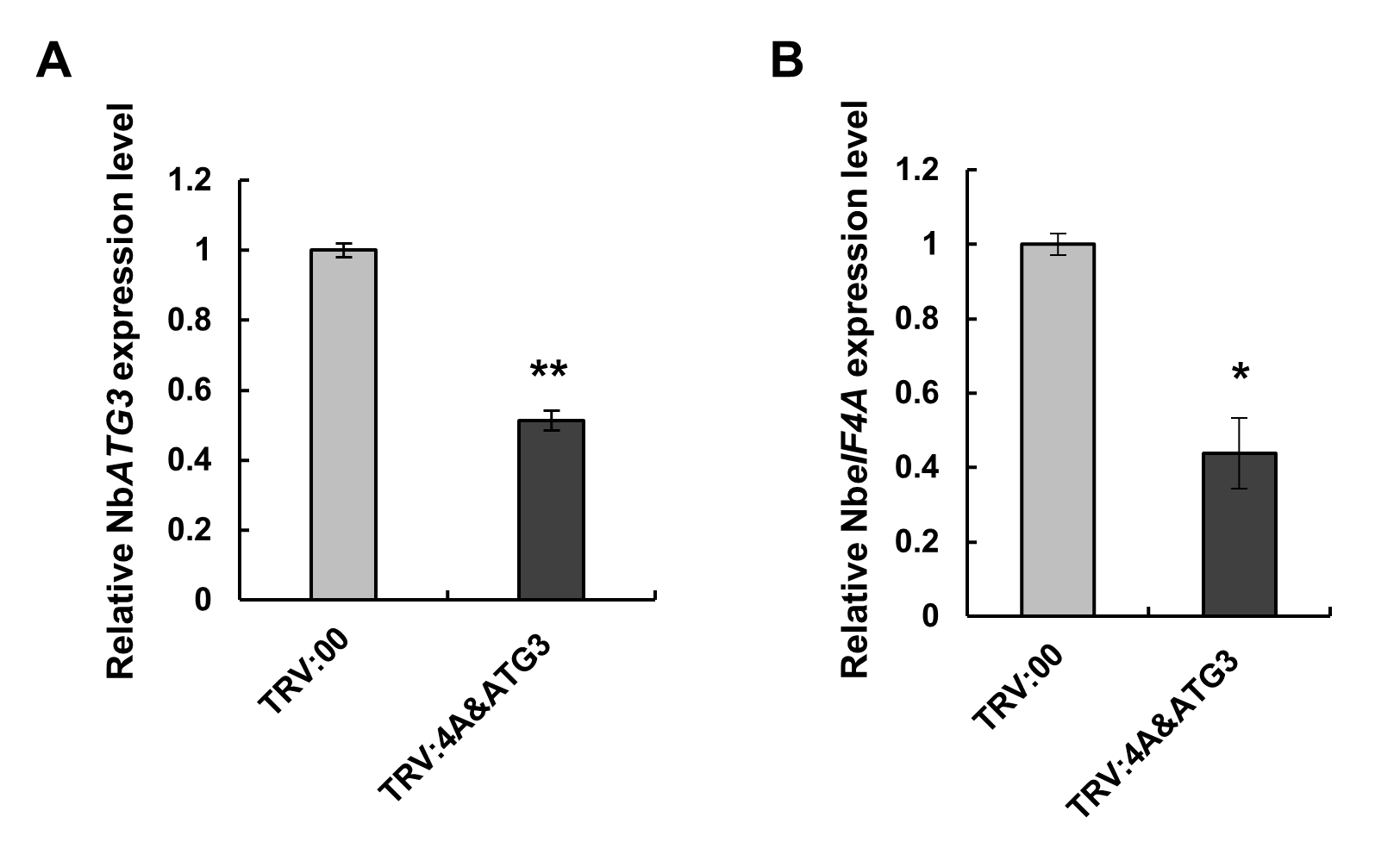

Supplement: S5 Fig — (A) Relative expression level of NbATG3 in NbATG3- and NbeIF4A co-silenced N. benthamiana plants. (B) Relative expression level of NbeIF4A in NbATG3 and NbeIF4A co-silenced N. benthamiana plants, as determined by RT-qPCR. NbActin served as the internal control. Error bars indicate standard error from three individual experiments. Asterisks indicate significant differences by Student’s t-test compared to the control (*, p<0.05; **, p<0.01). (TIF) [file ppat.1009963.s005.TIF]

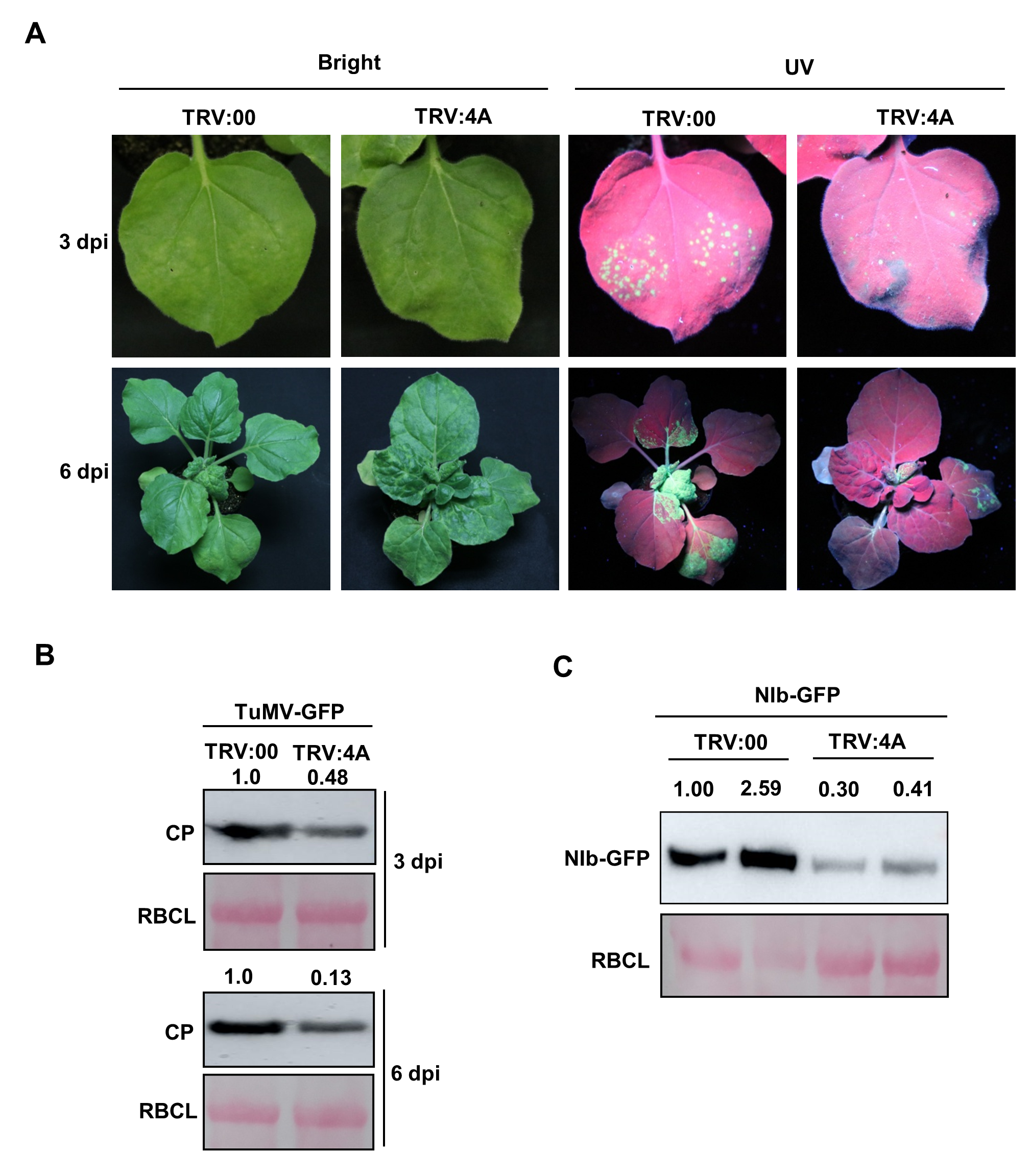

Supplement: S6 Fig — (A) TuMV-GFP infection in NbeIF4A-silenced (TRV:4A) plants. Non-silenced (TFV:00) plants were used as controls. In TuMV-GFP-inoculated leaves of NbeIF4A-silenced plants, the number of infection foci decreased significantly at 3 dpi. Fluorescent foci under UV illumination indicate GFP-positive infection foci. At 6 dpi, intensive fluorescence was observed in the top leaves of control plants, indicating the systemic infection of plants by TuMV-GFP. Weak fluorescence appeared in the top leaves of NbeIF4A-silenced plants. (B) Immunoblot analysis of TuMV-GFP accumulation in TRV:00- and TRV:4A-infected N. benthamiana plants with antibody of TuMV CP. (C) Immunoblot analysis of the accumulation of GFP-fused TuMV NIb protein transiently expressed in NbeIF4A-silenced and control leaves by Agrobacterium-mediated infiltration at 2.5 dpi. Band intensity was analyzed by ImageJ. (TIF) [file ppat.1009963.s006.TIF]

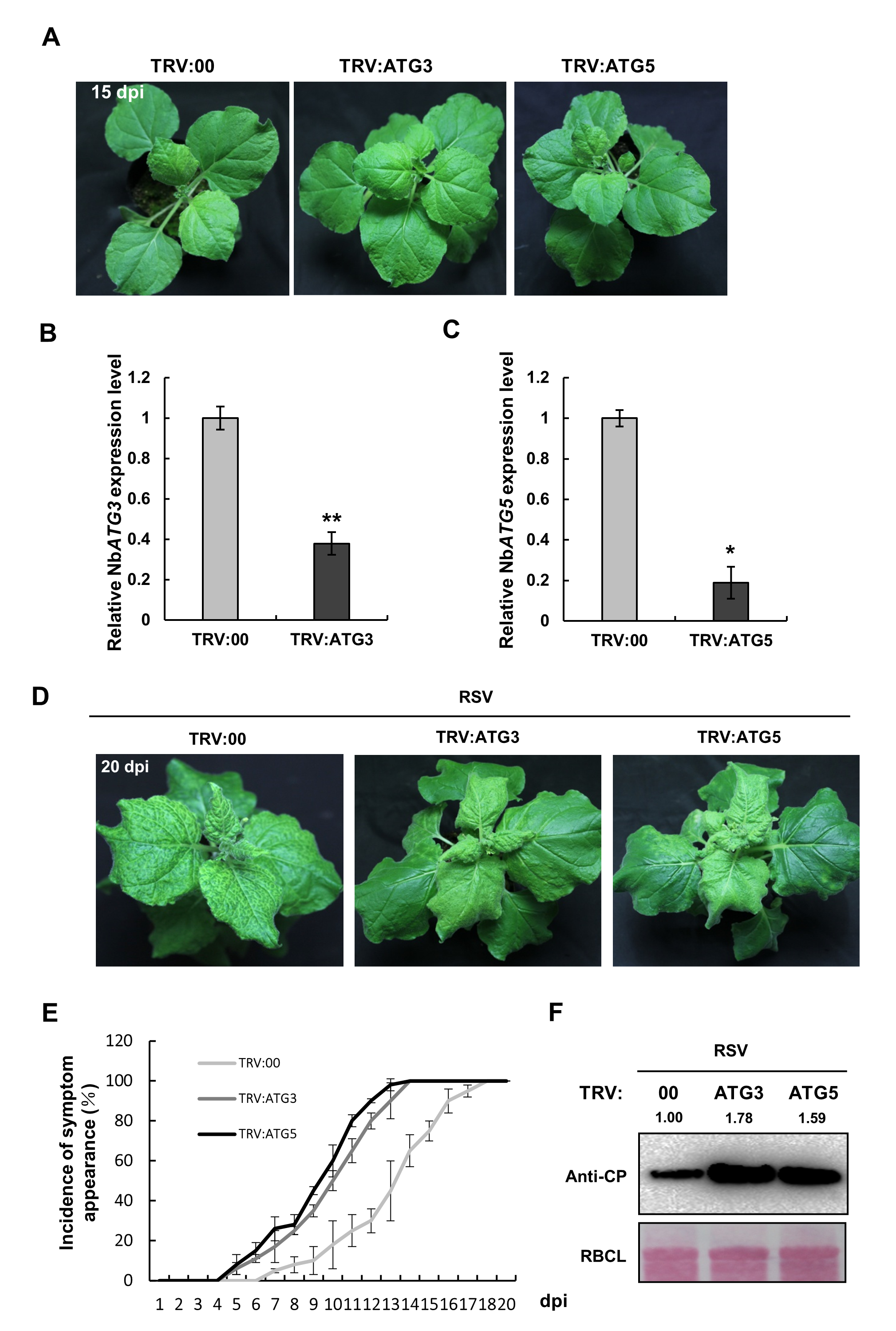

Supplement: S7 Fig — (A) Silencing of NbATG3 and NbATG5 individually did not cause obvious phenotypes in N. benthamiana. (B, C) Decreased expression of NbATG3 and NbATG5 in the corresponding silenced plants, as revealed by RT-qPCR, indicating the effective silencing of these genes. NbActin served as the internal control. Error bars indicate standard error from three individual experiments. Asterisks indicate significant differences by Student’s t-test compared to the control (*, p<0.05; **, p<0.01). (D) RSV symptoms in NbATG3-or NbATG5-silenced plants at 20 dpi. (E) Systemic RSV infection in silenced plants and control plants. Error bars indicate standard error from three individual experiments. Twenty plants were used for each experiment. (F) Immunoblot analysis of RSV accumulation in silenced plants. Band intensity was analyzed by ImageJ. (TIF) [file ppat.1009963.s007.TIF]

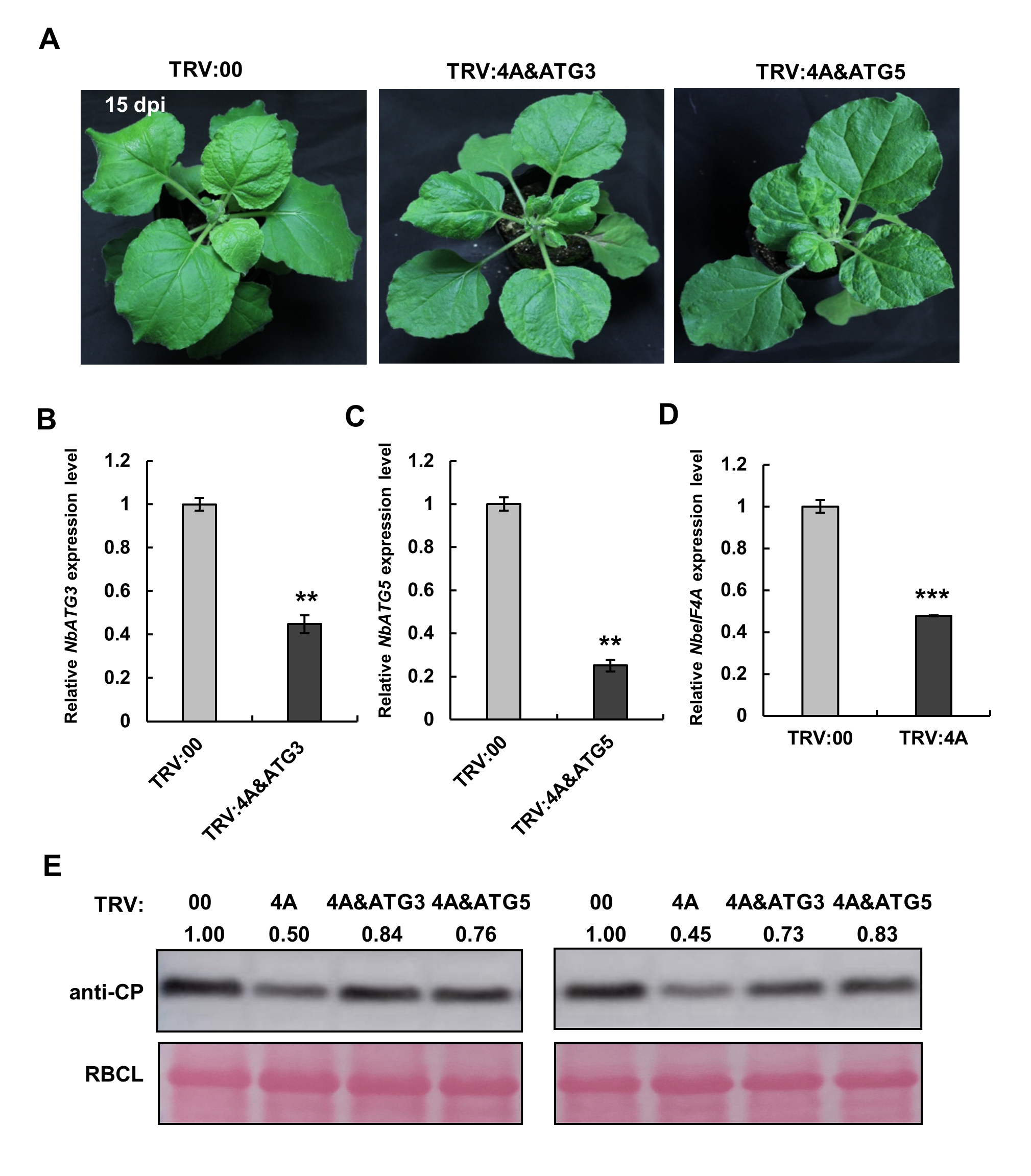

Supplement: S8 Fig — (A) Phenotypes of NbATG2, NbATG3, or NbATG5 and NbeIF4A co-silenced plants. (B–D) Decreased expression levels of NbATG3 (B), NbATG5 (C) and NbeIF4A (D) in the corresponding co-silenced plants, as revealed by RT-qPCR, indicating the efficient silencing of these genes. NbActin served as the internal control. Error bars indicate standard error from three individual experiments. Asterisks indicate significant differences by Student’s t-test compared to the control (*, p<0.05; **, p<0.01). (E) Immunoblot analysis of RSV CP accumulation levels in the infected plants in the other two repeated experiments. Band intensity was analyzed by ImageJ. (TIF) [file ppat.1009963.s008.TIF]

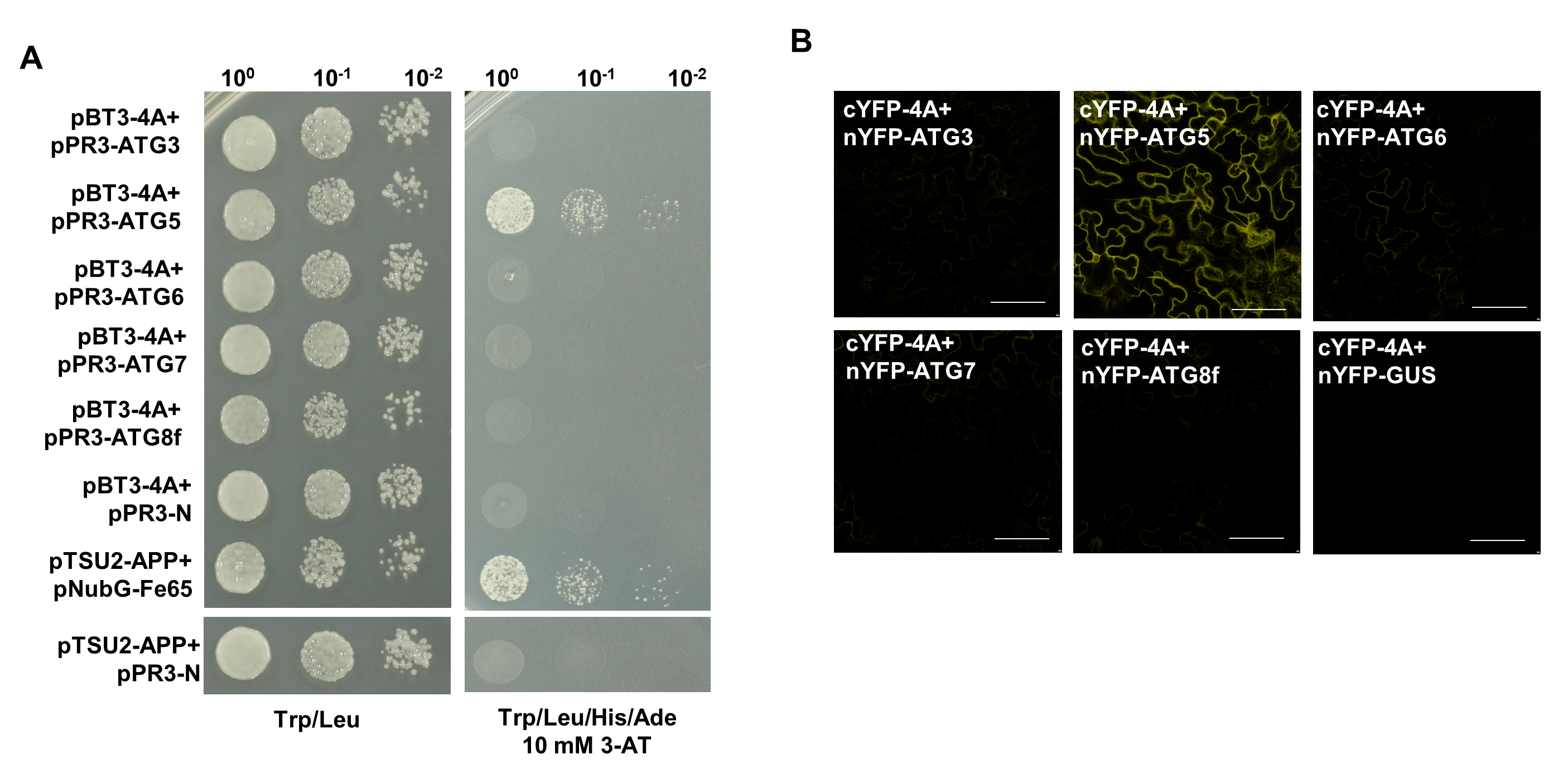

Supplement: S9 Fig — (A) NbATG5, but not other ATGs, interacted with NbeIF4A. The samples were plated onto selective medium lacking tryptophan and leucine (SD/-Trp/-Leu) and tryptophan, leucine, histidine, and adenine (SD/-Trp/-Leu/-His/-Ade) with 10 mM 3-AT. (B) BiFC assay confirming the results from Y2H. Bars, 20 μm. (TIF) [file ppat.1009963.s009.TIF]

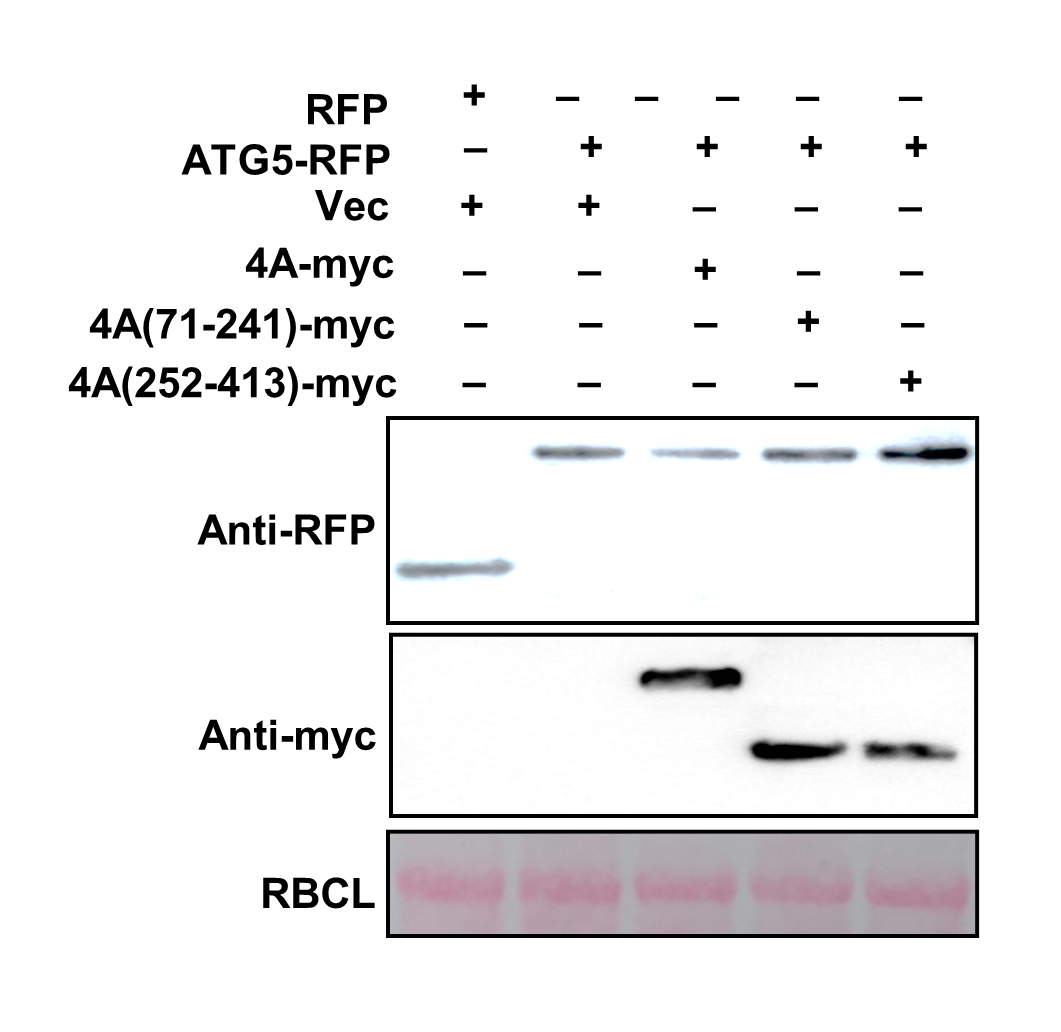

Supplement: S10 Fig — Expression of proteins used in the analysis of NbeIF4A-mediated inhibition of ATG5-activated autophagy was demonstrated using antibodies to the corresponding tags. (TIF) [file ppat.1009963.s010.TIF]

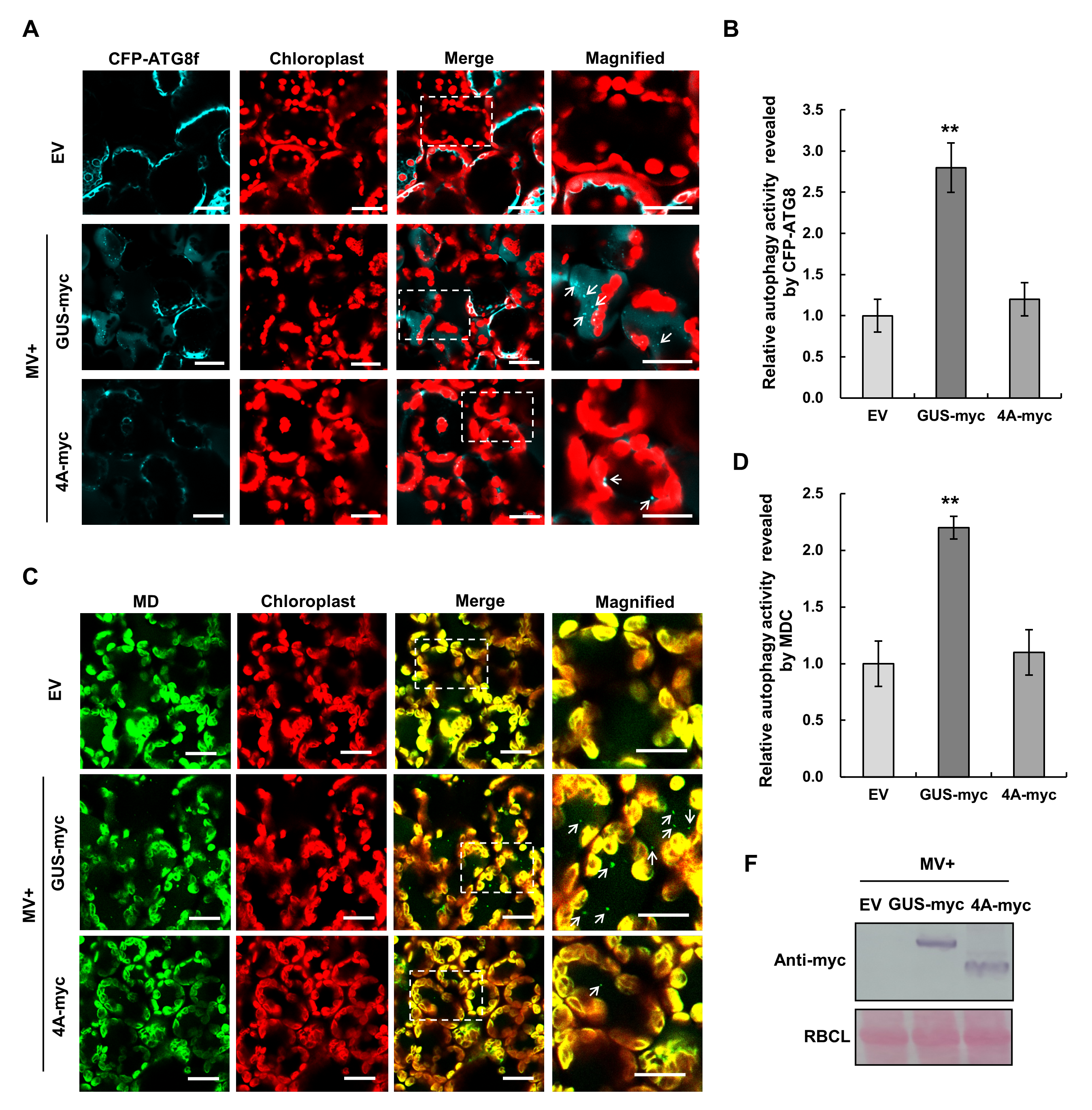

Supplement: S11 Fig — (A, C) Representative confocal images of dynamic autophagic activity observed in MV-treated cells, empty vector control (EV), and myc-tagged NbeIF4A-expressing cells, revealed by the specific autophagy marker CFP-NbATG8f (A) or MDC staining (C). Cyan: CFP-NbATG8f; green: MDC-stained structures; red: chlorophyll autofluorescence. Bars, 10 μm. (B, D) Relative autophagic activity in treated cells, normalized to that in EV cells, which was set to 1.0. Quantification of CFP-NbATG8f-labeled (B) or MDC-stained (D) autophagic foci per cell was performed by counting autophagic bodies to calculate autophagic activity. Over 150 cells per treatment were used for quantification. Bars indicate standard error from three individual experiments. Asterisks indicate significant differences by Student’s t-test compared to the control (**, p<0.01). (TIF) [file ppat.1009963.s011.TIF]

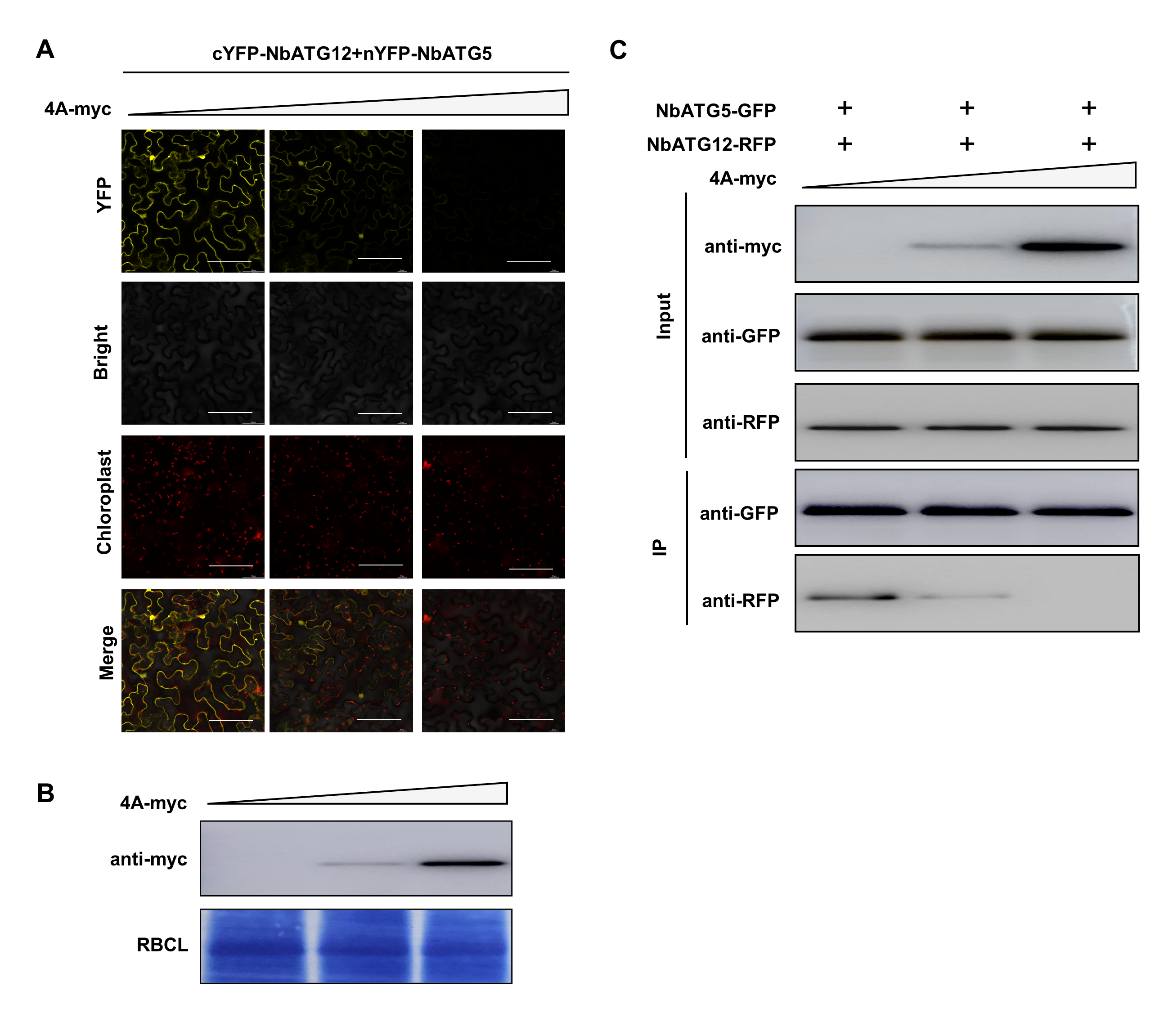

Supplement: S12 Fig — (A and B) NbATG5 interacted with NbATG12 in a BiFC assay, while the interaction signals were reduced by NbeIF4A expression (4A-myc). Bars, 20 μm. (C) In a Co-IP assay, when NbeIF4A was expressed, NbATG5 was immunoprecipitated, but it was difficult to immunoprecipitate NbATG12. (TIF) [file ppat.1009963.s012.TIF]

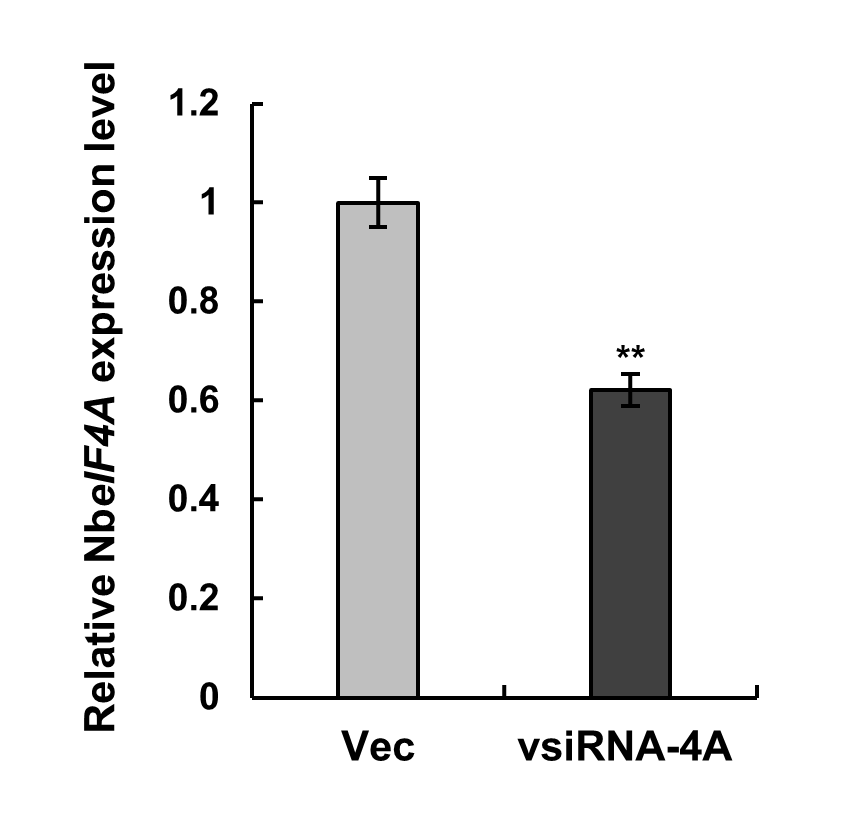

Supplement: S13 Fig — The relative transcript level of endogenous NbeIF4A was measured in cells where vsiRNA-4A was expressed via artificial miRNA expression. An unrelated small RNA (UUAAGGGUAAGUUUUCCGCAU) expressed in the vector (Vec) was used as the control. (TIF) [file ppat.1009963.s013.TIF]

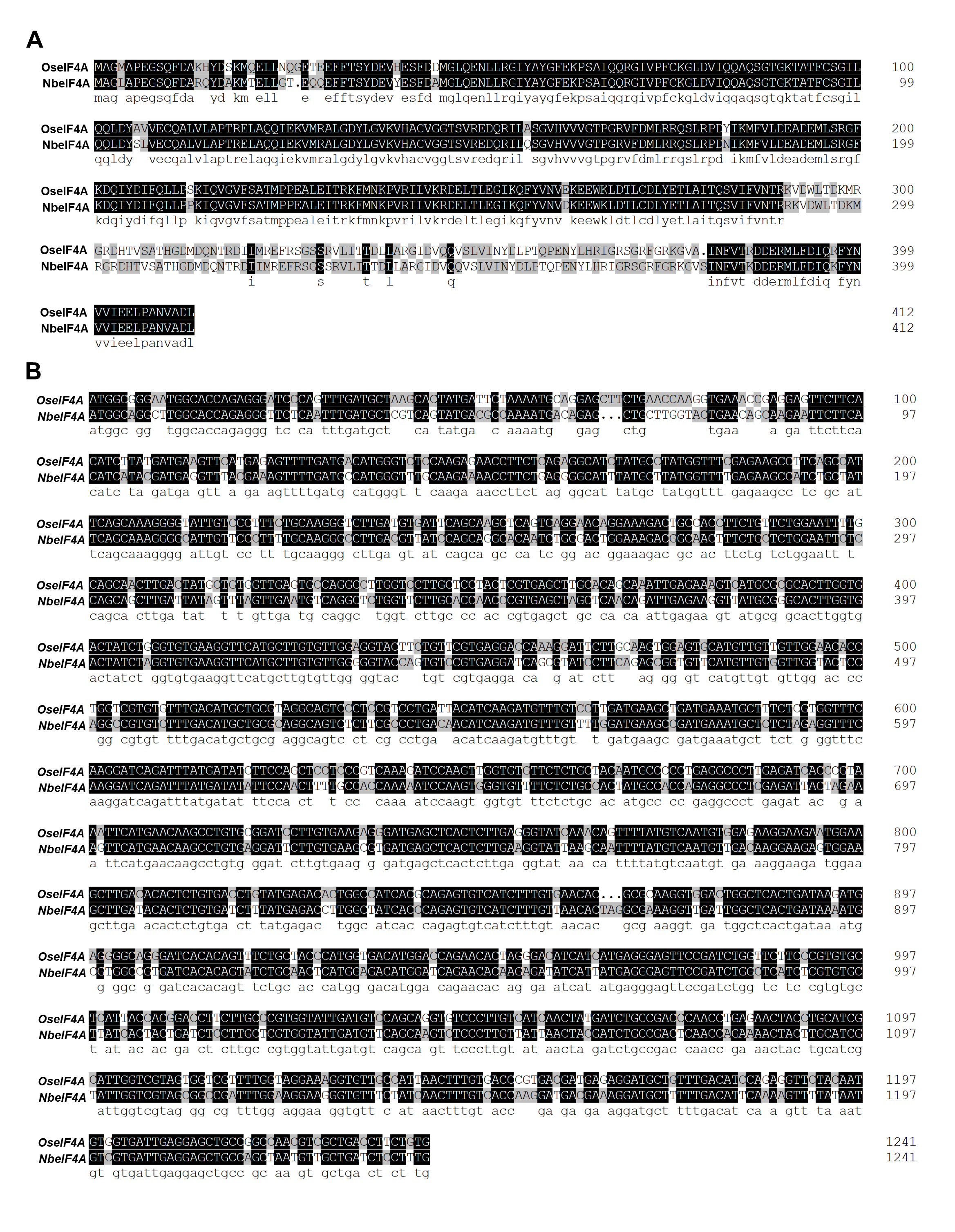

Supplement: S14 Fig — Amino acid (A) and nucleotide (B) sequence alignments. (TIF) [file ppat.1009963.s014.TIF]

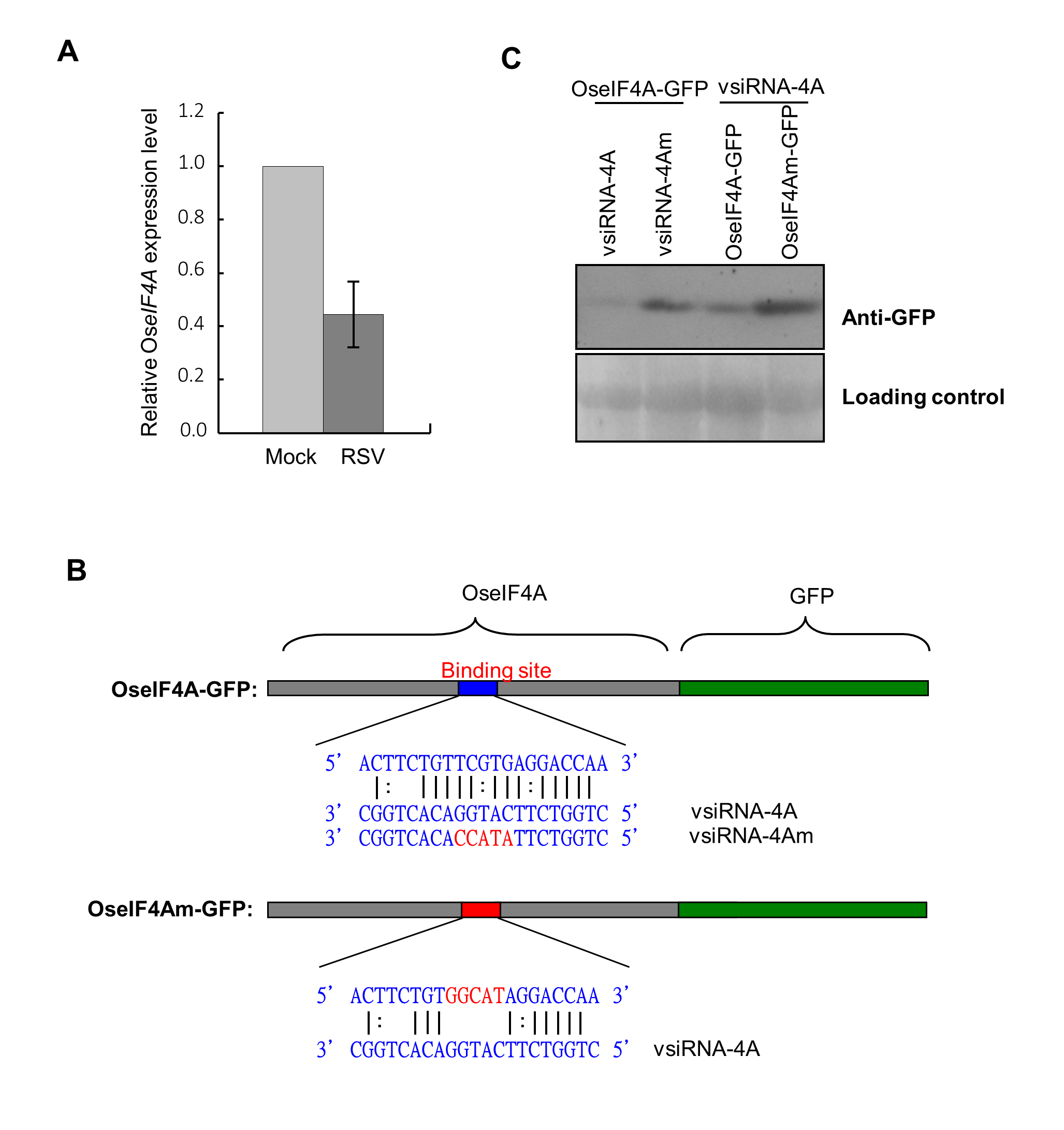

Supplement: S15 Fig — (A) OseIF4A transcript levels in RSV-infected rice at 14 dpi. (B) Immunoblot analysis using GFP-tagged OseIF4A (OseIF4A-GFP). For the control, a similar construct was prepared using a mutant OseIF4A (OseIF4Am-GFP) in which nucleotides 9–13 at the binding site of vsiRNA-4A were complementary to the wild type. vsiRNA-4Am represents the vsiRNA-4A mutant at nucleotides 9–13 (nucleotides in red). (C) GFP-tagged OseIF4A accumulation when co-expressed with vsiRNA-4A and in the controls (vsiRNA-4A/OseIF4Am-GFP or vsiRNA-4Am/OseIF4A-GFP) at 5 dpi. (TIF) [file ppat.1009963.s015.TIF]

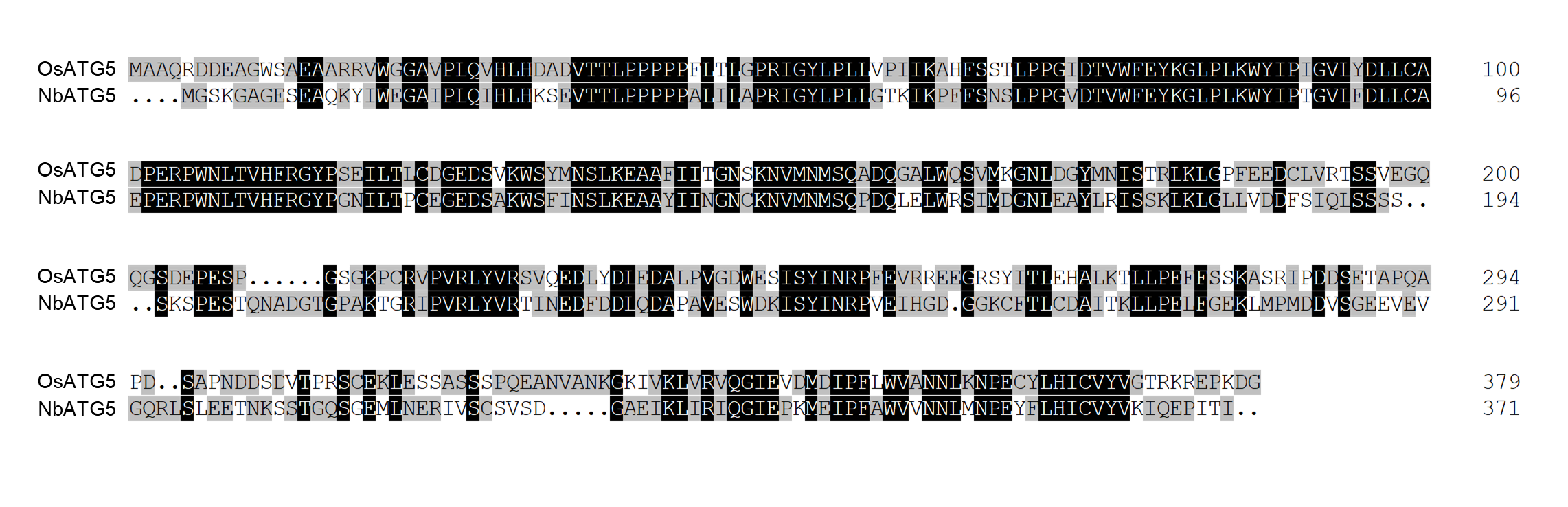

Supplement: S16 Fig — (TIF) [file ppat.1009963.s016.TIF]

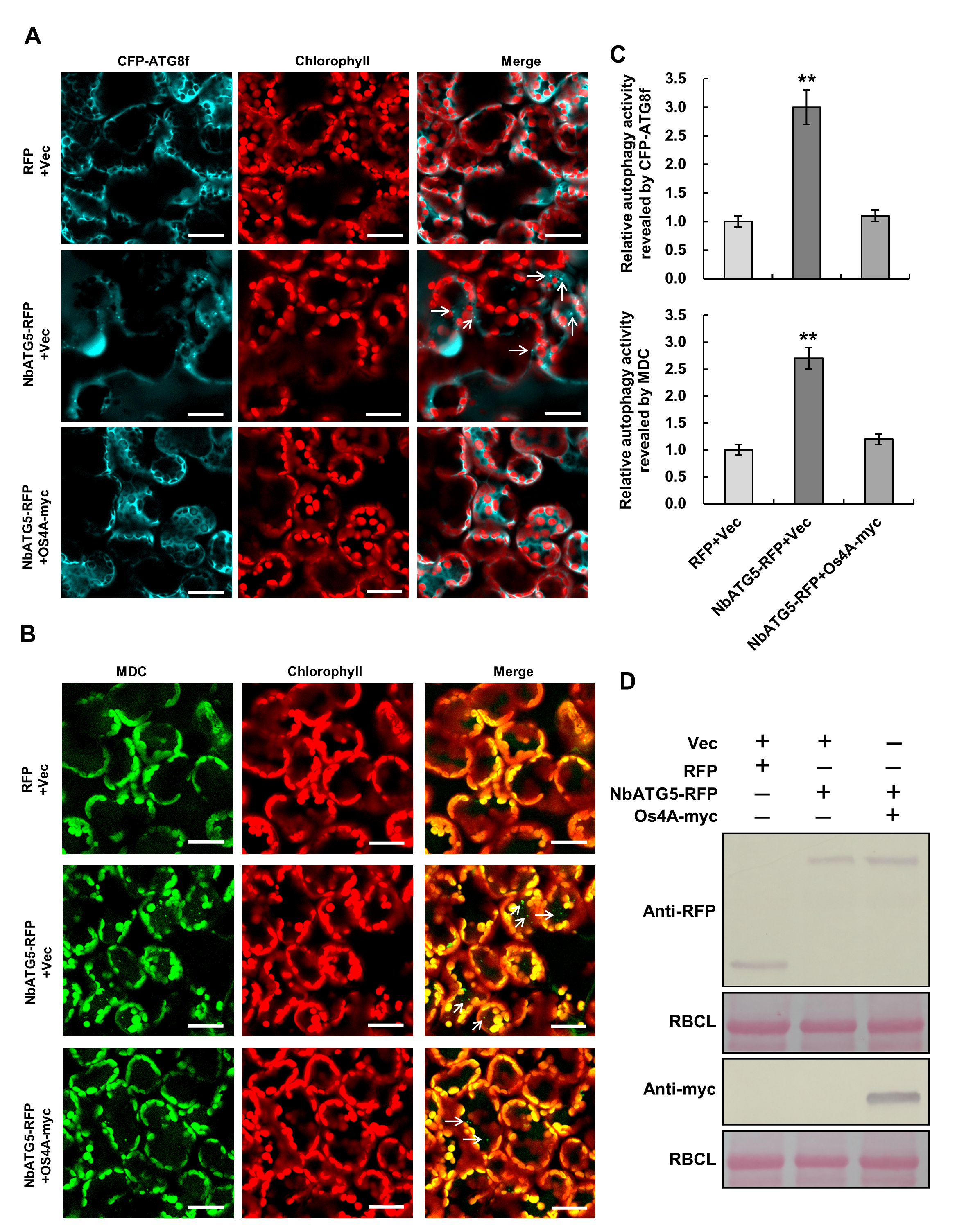

Supplement: S17 Fig — (A, B) Representative confocal images of autophagic activity revealed by NbATG8f-CFP (A) or MDC staining (B). (C) Relative autophagic activity, normalized to that of cells expressing RFP and empty vector. Quantification of autophagic foci per cell was performed by counting autophagic bodies to calculate autophagic activity. More than 150 cells per treatment were used for quantification. Error bars indicate standard error from three individual experiments. Asterisks indicate significant differences by Student’s t-test (**, p < 0.01) compared to the control. (D) Immunoblot analysis of protein accumulation. (TIF) [file ppat.1009963.s017.tif]
