# Supplementary material for: eIF4A, a target of siRNA derived from rice stripe virus, negatively regulates antiviral autophagy by interacting with ATG5 in Nicotiana benthamiana
Source: PLoS Pathog. 2021 Sep 29;17(9):e1009963. doi: 10.1371/journal.ppat.1009963 (PMC8504976; doi:10.1371/journal.ppat.1009963)
Supplement: S1 Table — (DOCX) [file ppat.1009963.s018.docx]

Table S1. Primers Used for Analysis

| **Prime name** | **Sequence (5’ – 3’)** |
| --- | --- |
| Y2H-NbATG3-F | CGACGACAAGACCGTCACCATGGTACTGTCACAGAAGATTCACG |
| Y2H-NbATG3-R | GAGGAGAAGAGCCGTCGCTATTCTGAAGCAGACCCATTGGGCTTG |
| Y2H-NbATG5-F | CGACGACAAGACCGTCACCATGGGAAGTAAAGGGGCAGGAG |
| Y2H-NbATG5-R | GAGGAGAAGAGCCGTCGCTATATGGTGATGGGTTCTTGAATTTTGAC |
| Y2H-NbATG6-F | CGACGACAAGACCGTCACCATGACGAAAAATAGCAGCAGTAGTAC |
| Y2H-NbATG6-R | GAGGAGAAGAGCCGTCGTCAAGATTGAAACTTGGTATTAGTTGGTTG |
| Y2H-NbATG7-F | CGACGACAAGACCGTCACCATGGCGGATAGTGGAAGAGGAAC |
| Y2H-NbATG7-R | GAGGAGAAGAGCCGTCGTTATATTTCTATAGAGTCATCGTCGTCCTTATC |
| Y2H-NbATG8f-F | CGACGACAAGACCGTCACCATGGCAAAGAGTTCATTCAAGC |
| Y2H-NbATG8f-R | GAGGAGAAGAGCCGTCGTTACACCAAGTTAAAGTCCCCAAATG |
| Y2H-NbeIF4A-F | CGACGACAAGACCGTCACCATGGCAGGCTTGGCACCAGAG |
| Y2H-NbeIF4A-R | GAGGAGAAGAGCCGTCGTCAAAGGAGATCAGCAACATTAGCTGGC |
| BiFC-NbATG3-F | CGACGACAAGACCGTCACCATGGTACTGTCACAGAAGATTCACG |
| BiFC-NbATG3-R | GAGGAGAAGAGCCGTCGTTCTGAAGCAGACCCATTGGGCTTG |
| BiFC-NbATG5-F | CGACGACAAGACCGTCACCATGGGAAGTAAAGGGGCAGGAG |
| BiFC-NbATG5-R | GAGGAGAAGAGCCGTCGTATGGTGATGGGTTCTTGAATTTTGAC |
| BiFC-NbATG6-F | CGACGACAAGACCGTCACCATGACGAAAAATAGCAGCAGTAGTAC |
| BiFC-NbATG6-R | GAGGAGAAGAGCCGTCGAGATTGAAACTTGGTATTAGTTGGTTG |
| BiFC-NbATG7-F | CGACGACAAGACCGTCACCATGGCGGATAGTGGAAGAGGAAC |
| BiFC-NbATG7-R | GAGGAGAAGAGCCGTCGTATTTCTATAGAGTCATCGTCGTCCTTATC |
| BiFC-NbATG8f-F | CGACGACAAGACCGTCACCATGGCAAAGAGTTCATTCAAGC |
| BiFC-NbATG8f-R | GAGGAGAAGAGCCGTCGCACCAAGTTAAAGTCCCCAAATG |
| BiFC-NbeIF4A-F | CGACGACAAGACCGTCACCATGGCAGGCTTGGCACCAGAG |
| BiFC-NbeIF4A-R | GAGGAGAAGAGCCGTCGAAGGAGATCAGCAACATTAGCTGGC |
| BiFC-NtATG12-F | CGACGACAAGACCGTCACCATGGCCTCCGATTCTCGTAAAGTGATTG |
| BiFC-NtATG12-R | GAGGAGAAGAGCCGTCGCTAACCGTTTCCTGCATTGTACAGGTC |
| BiFC-NbeIF4A(∆40-68)-F-1 | CGACGACAAGACCGTCACCATGGCAGGCTTGGCACCAGAG |
| BiFC-NbeIF4A(∆40-68)-R-2 | CTTGCAAAAGGGAACAATGCCCCTTTCGTAAACCTCATCGTATGATGTG |
| BiFC-NbeIF4A(∆40-68)-F-3 | CACATCATACGATGAGGTTTACGAAAGGGGCATTGTTCCCTTTTGCAAG |
| BiFC-NbeIF4A(∆40-68)-R-4 | GAGGAGAAGAGCCGTCGAAGGAGATCAGCAACATTAGCTGGC |
| BiFC-NbeIF4A(71-241)-F | CGACGACAAGACCGTCACCGTTCCCTTTTGCAAGGGCCTTGACG |
| BiFC-NbeIF4A(71-241)-R | GAGGAGAAGAGCCGTCGAATCCTCACAGGCTTGTTCATGAAC |
| BiFC-NbeIF4A(252-413)-F | CGACGACAAGACCGTCACCATTAAGCAATTTTATGTCAATGTTGACAAG |
| BiFC-NbeIF4A(252-413)-R | GAGGAGAAGAGCCGTCGAAGGAGATCAGCAACATTAGCTGGC |
| BiFC-NbeIF4A(71-187)-F | CGACGACAAGACCGTCACCGTTCCCTTTTGCAAGGGCCTTGACG |
| BiFC-NbeIF4A(71-187)-R | GAGGAGAAGAGCCGTCGAACAAACATCTTGATGTTGTCAGGGC |
| BiFC-NbeIF4A(195-241)-F | CGACGACAAGACCGTCACCTCTAGAGGTTTCAAGGATCAG |
| BiFC-NbeIF4A(195-241)-R | GAGGAGAAGAGCCGTCGAATCCTCACAGGCTTGTTCATGAAC |
| BiFC-NbeIF4A(∆187-195)-F-1 | CGACGACAAGACCGTCACCATGGCAGGCTTGGCACCAGAG |
| BiFC-NbeIF4A(∆187-195)-R-2 | CTGATCCTTGAAACCTCTAGAAACAAACATCTTGATGTTGTCAGGGCG |
| BiFC-NbeIF4A(∆187-195)-F-3 | CGCCCTGACAACATCAAGATGTTTGTTTCTAGAGGTTTCAAGGATCAG |
| BiFC-NbeIF4A(∆187-195)-R-4 | GAGGAGAAGAGCCGTCGAAGGAGATCAGCAACATTAGCTGGC |
| OE-NbATG5-F | CGACGACAAGACCGTCACCATGGGAAGTAAAGGGGCAGGAG |
| OE-NbATG5-R | GAGGAGAAGAGCCGTCGTATGGTGATGGGTTCTTGAATTTTGAC |
| OE-NbATG8f-F | CGACGACAAGACCGTCACCATGGCAAAGAGTTCATTCAAGC |
| OE-NbATG8f-R | GAGGAGAAGAGCCGTCGTTACACCAAGTTAAAGTCCCCAAATG |
| OE-NbeIF4A-F | CGACGACAAGACCGTCACCATGGCAGGCTTGGCACCAGAG |
| OE-NbeIF4A-R | GAGGAGAAGAGCCGTCGAAGGAGATCAGCAACATTAGCTGGC |
| OE-p3-XbaI-F | TCTAGAATGAACGTGTTCACATCGTCT |
| OE-p3-KpnI-R | GGTACCCAGCACAGCTGGAGAGCTG |
| OE-p3IP-KpnI-F | GGTACCATGGAGGGATTATCAGCATCA |
| OE-p3IP-BamHI-R | GGATCCCGGTTGGCACATAATCTCATT |
| OE-NbeIF4A(71-241)-F | CGACGACAAGACCGTCACCGTTCCCTTTTGCAAGGGCCTTGACG |
| OE-NbeIF4A(71-241)-R | GAGGAGAAGAGCCGTCGAATCCTCACAGGCTTGTTCATGAAC |
| OE-NbeIF4A(252-413)-F | CGACGACAAGACCGTCACCATTAAGCAATTTTATGTCAATGTTGACAAG |
| OE-NbeIF4A(252-413)-R | GAGGAGAAGAGCCGTCGAAGGAGATCAGCAACATTAGCTGGC |
| OE-NtATG12-F | CGACGACAAGACCGTCACCATGGCCTCCGATTCTCGTAAAGTGATTG |
| OE-NtATG12-R | GAGGAGAAGAGCCGTCGCTAACCGTTTCCTGCATTGTACAGGTC |
| RSV-cp-F | ATGGGCACCAACAAGCCAGCCAC |
| RSV-cp-R | CTAGTCATCTGCACCTTCTGCCTCG |
| nLUC-NbeIF4A-F | ACGAGCTCGGTACCCGGGATCCATGGCAGGCTTGGCACCAGAGGGT |
| nLUC-NbeIF4A-R | GACGCGTACGAGATCTGGTCGACAAGGAGATCAGCAACATTAGC |
| cLUC-NbATG5-F | GTACGCGTCCCGGGGCGGTACCATGGGAAGTAAAGGGGCAGGAG |
| cLUC-NbATG5-R | GAACGAAAGCTCTGCAGGTCGACCTATATGGTGATGGGTTCTTG |
| TRV:4A&ATG3-F-1 | CGACGACAAGACCGTCACCACTGCTGCCACCAAAGATTCAAG |
| TRV:4A&ATG3-R-2 | GTGAATCTTCTGCGACAGTAGCAGATACTGTATGATCACGGCTG |
| TRV:4A&ATG3-F-3 | CAGCCGTGATCATACAGTATCTGCTACTGTCGCAGAAGATTCAC |
| TRV:4A&ATG3-R-4 | GAGGAGAAGAGCCGTCGCTTTCTTTAGGTTTCCCATG |
| TRV:4A&ATG5-F-1 | CGACGACAAGACCGTCACCACTGCTGCCACCAAAGATTCAAG |
| TRV:4A&ATG5-R-2 | CCCTGAATGCGTATGAGCTTGCAGATACTGTATGATCACGGCTG |
| TRV:4A&ATG5-F-3 | CAGCCGTGATCATACAGTATCTGCAAGCTCATACGCATTCAGGG |
| TRV:4A&ATG5-R-4 | GAGGAGAAGAGCCGTCGGCTTCGGACCTTTGCTACCT |
| NbeIF4A-hairpin-F | GGATCCATGGGTCAATGTTGACAAGGAAGAGTGGAAG |
| NbeIF4A-hairpin-R | TCTAGACTCGAGCGGCAGATCGTAGTTAATAAC |
| NbeIF6A-hairpin-F | CGCGGATCCATGG CCATTGAAGACTTGGACGAAC |
| NbeIF6A-hairpin-R | CTAGTCTAGACTCGAG CCTCATCTCATCAACAATAGCA |
| TRV:NbeIF4A-F | ATGCTTATGGTTTTGAGAAGCC |
| TRV:NbeIF4A-R | TAAATCTGATCCTTGAAACCTCTAGAG |
| TRV:NbATG3-F | CTTGGTCTTGGGAATCAGGTGAAC |
| TRV:NbATG3-R | CCTCATTATCAAGCAGAACTTCACCTC |
| TRV:NbATG5-F | CACCTTGTGAAGGAGAAGATAGTGC |
| TRV:NbATG5-R | CCGGTATTCTACCTGTTTTAGCTGG |
| NbATG2-qRCR-F | GCAATTGGGCTTGGAGTGCATTTG |
| NbATG2-qRCR-R | CCTGTCGGGCATCTCTAGGTTGAT |
| NbATG3-qRCR-F | GGAGGTGAGGAGGAGGAAGATATTCCAG |
| NbATG3-qRCR-R | CAGGCTCATGAGCCACAAGATATGC |
| NbATG5-qRCR-F | CTGATGCCAATGGATGACGTTTCTGG |
| NbATG5-qRCR-R | CCATCTGACACGGAGCAGGAGAC |
| NbATG6-qRCR-F | ACCTGCGTAAAGGAGTTTGCTGAC |
| NbATG6-qRCR-R | AGAGCTTTGGTCCAACTTTCCTGC |
| NbATG7-qRCR-F | CCAGCAGTGGAAGCAGAAGGTCTT |
| NbATG7-qRCR-R | GCCACCGACTTTCCCGTGTATCA |
| NbPI3K-qRCR-F | GCTGTGCTGGTTACTCCGTCATC |
| NbPI3K-qRCR-R | ACTGACTTTCCGCTCCACCCATA |
| NbACTIN-qRCR-F | CCCAGAGAGGAAATACAGTG |
| NbACTIN-qRCR-R | CAATAGACGGACCAGATTCG |
| NbeIF4A-qRCR-F | GGTCGTAGCGGCCGATTTGGAAGG |
| NbeIF4A-qRCR-R | GCAACATTAGCTGGCAGCTCCTC |
| NbeIF6A-qRCR-F | GGTTCCTCTTGTTGCGGGAAC |
| NbeIF6A-qRCR-R | CAATAACGGACAACTCTGTAGCAG |
| TRV-NbATG3-qRCR-F | GACGAGGATGACAACTTGCCATCAATG |
| TRV-NbATG3-qRCR-R | GTTCATCGTACTCTCCCATGTCTGG |
| TRV-NbATG5-qRCR-F | GATGACGTTTCTGGAGAGGAAGTTG |
| TRV-NbATG5-qRCR-R | CTCAGCACCATCTGACACGGAGC |
| TRV-NbeIF4A-qRCR-F | GAGGCCCTCGAGATTACTAGAAAGTTC |
| TRV-NbeIF4A-qRCR-R | CCACTCTTCCTTGTCAACATTGACAT |
| RSV-cp-qRCR-F | GCTGGAAGCACAGAGGCTTCGAC |
| RSV-cp-qRCR-R | CTGAGCCAGCCTTCCAAGTGTG |
