# Supplementary material for: eIF4A, a target of siRNA derived from rice stripe virus, negatively regulates antiviral autophagy by interacting with ATG5 in Nicotiana benthamiana
Source: PLoS Pathog. 2021 Sep 29;17(9):e1009963. doi: 10.1371/journal.ppat.1009963 (PMC8504976; doi:10.1371/journal.ppat.1009963)
Supplement: Attachment — Submitted filename: Response-2021.06.20-Edited-FY.doc [file ppat.1009963.s019.doc]

We thank Dr. Nagy and the reviewers for their constructive comments on our manuscript. In response to the comments and suggestions, we have modified our manuscript and corrected the errors. In the text below, the comments are shown in black and our replies are shown in red.

**Part I - Summary**

Editorial comments: As you see from the reviews, reviewers raise major concerns about the findings reported in this manuscript overearlier work. Specifically, previous work showing silencing impact RSV infection and p3 of RSV is targeted for degradation through autophagy; the mechanistic relationship between eIF4A and autophagy described here is unclear. Considering silencing of eIF4A will have general impact on translation, how specific is the observed effect on RSV and autophagy? Furthermore, specific experiments that led to the identification of eIF4A interaction with ATG5 are lacking. Does eIF4A only interact with ATG5; if so, what is the biochemical consequence of this interaction on autophagy and how does this impact RSV infection? Reviewers also raise concerns about the quality of images regarding RSV symptoms, protein degradation assays and autophagy.

Reply: We thank Dr. Nagy for the constructive comments. We have performed additional experiments to clarify the mechanistic relationship between eIF4A and autophagy. The results showed that eIF4A competes with ATG12 for interactions with ATG5, hence interfering with the interaction between ATG5 and ATG12, shedding light on a mechanism by which eIF4A inhibits autophagy. We also examined whether silencing of *eIF4A* would affect general translation. Silencing of *eIF4A* did not significantly affect translation. However, in the positive control, silencing of *eIF6A*, a key component of the translational machinery, suppressed translation significantly, suggesting that the effect of silencing *eIF4A* on autophagy was not due to the translational machine. Moreover, we added information on the identification of ATG5 and improved the quality of the images in the figures.

Reviewer #1: This paper describes the novel function of eukaryotic translation

initiation factor 4A (eIF4A) as a negative regulator of autophagy in Nb and rice

possibly through interaction with ATG5, a key component of autophagosome

biogenesis. The authors provide evidences that a virus-derived small interfering RNA

originated from rice stripe virus (RSV) RNA4 cleaves the eIF4A transcript of Nb,

resulting in activation of autophagy. The results are convincing and most of the

conclusions are supported by presented data, except that I think that figures 2, 5, 6 and

7 are important but at the resolutions shown it is not sufficient to definitively see the

autophagic fluorescent foci.

Reply: We appreciate the reviewer’s useful comments and apologize for the poor quality of the figures in the original manuscript. In the revised version of the manuscript, the quality of the images has been improved (Please download high resolution figures for reviewing).

Reviewer #2: This study may present interesting findings that link authors’ previous studies and contribute to our knowledge and studies regarding antiviral immunity. However, so far, there are issues and lack of evidence that should be addressed/examined for publication.

1) A hypothesized model constructed according to authors’ this and previous studies has an apparent contradiction; RSV keeps the genomic sequence, from which derived vsiRNA silences eIF4A (on purpose) to induce antiviral autophagy. Authors have previously reported that vsiRNA derived from the infected RSV genomic RNA silences eIF4A, resulting in symptom expressions (Shi et al., New Phytologists, 209, 1106, 2016). Furthermore, in this study, RSV multiplication and its virulence reduced in N. benthamiana plants, in which eIF4A expression was silenced by VIGS. Following results in this study indicate eIF4A is a negative regulator of autophagy by interacting with and inhibiting ATG5. Authors recently reported that RSV p3, RNA silencing suppressor, is degraded by autophagy via interacting with NbP3IP-NbATG8 (Jiang et al., New Phytologists, on-line, 2020). These results and reports suggest that RSV apparently induces antiviral immunity on purpose by silencing eIF4A with vsiRNA since silencing of eIF4A inhibits RSV infection by inducing autophagy-mediated degradation of p3, resulting in reinforcement of antiviral RNA silencing. This reviewer thinks that RSV can easily evade the induction of eIF4A-silencing-mediated immunity by acquiring (nonsynonymous) mutations in the region, where vsiRNA to eIF4A is produced. However, RSV keeps the sequence, why? RSV has some advantages from silencing of eIF4A even though inducing antiviral autophagy? Can authors explain or discuss this point in Discussion section. -

Reply: We agree with the reviewer’s comment. Indeed, based on these results, it is difficult to understand why RSV retained a sequence that can induce autophagy. However, a recent study demonstrated that the movement protein NSvc4 of RSV induces the autophagic turnover of the group 1 remorin (REM1) protein to prevent the REM1-mediated negative regulation of viral cell-to-cell movement (Fu, et al. 2018. Rice Stripe Virus Interferes with S-acylation of Remorin and Induces Its Autophagic Degradation to Facilitate Virus Infection. Molecular Plant), which indicates that autophagy is also beneficial for RSV. We added this information to the text (Lines 392–394).

**Part II –Major Issues: Key Experiments Required for Acceptance**

Reviewer #1: None.

Reviewer #2:

2) Fig. 1 has many flaws though showing effect of silencing eIF4A on RSV infection is very important in this study. Authors described that three individual inoculation tests with each 20 eIF4A silenced and empty-TRV infected plants with RSV, indicating totally 120 plants (60 eIF4A-silenced and 60 empty-TRV infected plants) were inoculated with RSV. However, authors showed just one picture of each eIF4A-silencied/non-silenced with RSV and an RSV CP- immunoblot with just one sample. Authors described systemic infection with RSV was confirmed by RT-PCR. However, authors did not show any results of RT-PCR.

Reply: Thank you for the comments. The typical symptoms of RSV in *eIF4A*-silenced and non-silenced plants are shown in Figure 1. To further support the results with additional data, we provide the results of RT-PCR following RSV infection (in three replicates) in Fig. S2. We also describe CP accumulation in RSV-infected *eIF4A*-silenced plants (Lines 129–131).

Moreover, the legend of Fig. 1D says “RSV infection rate” but the bar graph says “plants with symptoms (%)”, which is true? Authors should clarify whether RSV can systemically infect eIF4A-silenced plants but its virulence is reduced (less symptoms) or RSV cannot infect the silenced plants systemically.

Reply: We apologize for the error. The figure indeed shows the RSV infection rate. We have corrected this error throughout the text. As mentioned in the Results, both the RSV infection rate and RSV accumulation in infected *eIF4A*-silenced plants decreased significantly, which indicates that both virulence and systemic infection of RSV were reduced. We added this information to the text (Line 131).

This inoculation test is a critical point in this study.Authors should show all of RT-PCR and RSV CP immunoblotting with 120 samples and statically analyze accumulation level of RSV CP to clarify whether RSV CP level is actually reduced in eIF4A-silenced plants.

Reply: Thank you for the comment. We have now added these results to Fig. S2.

3) In Fig. 1C, authors may want to show attenuated symptoms caused by RSV infection in eIF4A-silenced plants. However, according to the pictures, this reviewer thinks symptoms of the eIF4A-silenced plant with RSV are comparable or more severe than control. Show two or more pictures of each silenced/nonsilenced plants with RSV, showing different severity of symptoms, and let us verify this point.

Reply: Thank you for the comments. Silencing of *eIF4A* caused leaf-twisting and stunting at 14 dpi, which may have interfered with the observation of RSV symptoms. The most obvious symptom of RSV infection was yellow and mosaic leaves, which was attenuated in *eIF4A*-silenced plants. We added three pairs of photographs to the supplementary file to further show this difference (Fig. S1). We also describe this difference in detail in the text (Line 129. Fig. S1).

4) Although there are number of host factors involved in autophagy, readers would be interested in how authors screened and identified ATG5 as an interactor with eIF4A. However, authors just described “we tested the potential interaction between NbeIF4A and proteins with functions in autophagy by bimolecular fluorescence complementation (BiFC) assays.” How many and which ATGs did authors examined? How did authors screen candidates by BiFC? If the examined ATGs were limited numbers, how did authors select them? Answers should be described in the text.

Reply: We agree with this comment and have now added this information to the text. In the experiment, we investigated the interactions of eIF4A with ATG3, ATG5, ATG6, ATG7, and ATG8, which have been reported to function in the interplay between viruses and autophagy. Only ATG5 interacted with eIF4A. We added this information to the text (Lines 242–245).

5) Fig. 3A examined effect of eIF4A-silencing on accumulation of transiently expressed p3 by agroinfiltration and showed reduced accumulation of p3 in eIF4A-silenced plants, implying enhanced degradation of p3 by autophagy. However, this reviewer does not think this experiment worked. Transient expression of a certain protein by agroinfiltration is very unstable and dependent on conditions of leaf even in one plant. Therefore, effect of silencing or overexpression of a certain protein (eIF4A in this study) on expression or its activity level of a examined protein (p3 in this study) should be compared with control expressions in “one leaf”. However, in this manuscript, silencing of eIF4A was done with VIGS systemically. Thus, authors definitely used leaves of different plants to compare the p3 expression level between eIF4A-silenced and -nonsilenced conditions. For this experiments, authors should prepare a binary vector expressing dsRNA (inveted-repeat of eIF4A partial cDNA) to induce RNAi against eIF4A and co-agroinfiltrate with the p3-myc expression vector in order to see the effect of eIF4A silencing on p3 in one leaf. To confirm the effect of eIF4A silencing on p3 expression, authors should examine the effect on p3 RNA silencing suppressor activity with GFP too.

Reply: Thank you for the comments. We performed this experiment, as suggested. We developed a construct expressing the hairpin RNA of *eIF4A* and used it to analyze the effect of *eIF4A* silencing on p3 expression in one leaf vs. the control. Moreover, the plants in this analysis co-expressed GFP to reveal the effect of RNA silencing on the activity of the suppressor p3. These results were used to replace the original ones in Fig. 3A (Fig. 3A-C, Lines 205–213).

**Part III – Minor Issues: Editorial and Data Presentation Modifications**

Reviewer #1: 1. Page 5: “RT-PCR confirmed the systemic infection by RSV of plants with symptoms.” These data do not appear in the manuscript.

Reply: We have now provided the RT-PCR results of RSV infection in three replicates in Supplementary File 1. We also describe CP accumulation in RSV-infected eIF4A-silenced plants (Lines 129–131).

2. The signals in the immunoblot shown in Figure 3 are very weak making little bit hard to judge the results.

Reply: We repeated the experiment again and provided the immunoblot, which shows strong signals (Fig. 3F).

3. Although silencing of ATG3 is sufficient for canceling the effect of eIF4A- silencing on autophagy induction (Figure 2A), accumulations of RSV CP in eIF4A+ATG3 co-silenced plants are only 39% of control plants (Figure 3D). This suggests that eIF4A has another function during RSV infection in addition to inhibition of anti-viral autophagy.

Reply: We agree with this comment. In the experiments, which were repeated many times, silencing of *ATG3* was not sufficient to cancel the effect of *eIF4A* silencing on RSV accumulation, which indeed suggests that eIF4A may have another function during RSV infection. We added a discussion of this issue to the text (Lines 236–239).

4. Page 8: How did the authors identify NbATG5 as an interactor of NbeIF4A? Please include more details for this.

Reply: Agreed. We added this information to the text (Lines 242–245).

5. Page 10, line 3: Delete “the silencing of”.

Reply: Deleted.

6. According to the experimental data shown, eIF4A and ATG5 coimmunoprecipitated and are close proximity as observed by BiFC and split- Luc experiments (Figure 4 and 7). However, a “direct physical interaction” of eIF4A with ATG5 was not substantiated by the presented data.

Reply: Agreed. We revised this description, as suggested. (Fig. 4D).

7. How does the eIF4A- ATG5 interaction inhibit autophagy? Does eIF4A interfere the ATG5-ATG12 complex formation?

Reply: Thank you for the constructive comment. We performed an additional experiment to explore this issue. The results showed that the expression of eIF4A interfered with the interaction between ATG5 and ATG12, providing further information about the mechanism by which eIF4A inhibits autophagy. We added these results to the text (Fig. S12, Lines 282–292).

Reviewer #2: 5)Provide a general introduction of the eIF4A function and role in plants and so far, reported relationships of it with virus or pathogen infections or plant immunity in Introduction section.

Reply: Agreed. We added this information to the Introduction section (Lines 62-75).

6) This study implies a negative regulation of autophagy, degradation of proteins, by eIF4A, which is involved in translation or production of protein. Apart from antiviral defense, can authors discuss novel roles or contributions of eIF4A in gene-expression generally?

Reply: Agreed. We added this information to the Discussion section (Lines 348–357).

7) Minor points P6L12 and L29, Nib should be Nib

Reply: Corrected.
