# Supplementary material for: eIF4A, a target of siRNA derived from rice stripe virus, negatively regulates antiviral autophagy by interacting with ATG5 in Nicotiana benthamiana
Source: PLoS Pathog. 2021 Sep 29;17(9):e1009963. doi: 10.1371/journal.ppat.1009963 (PMC8504976; doi:10.1371/journal.ppat.1009963)
Supplement: Attachment — Submitted filename: Response to Reviewers.doc [file ppat.1009963.s020.doc]

We thank the reviewers for their constructive comments and have now modified the script to correct the errors and to respond to the comments and suggestions. In the text below, the comments received are in black and our replies in red.

Reviewer's Responses to Questions

**Part I - Summary**
Please use this section to discuss strengths/weaknesses of study, novelty/significance, general execution and scholarship.

Reviewer #1: Authors have carefully addressed most of the issues raised in the last review. I have just a couple of comments.

Reviewer #2: The authors well revised the manuscript by addressing issues that the editor and reviewers raised with additional or re-examined data. Although not wholly convinced yet with complex interactions among RSV, host RNA silencing, and autophagy described in this study, this reviewer agrees that this study is worth being published in terms of its suggesting multiple or concatenated interactions of viral vsiRNAs, silencing suppressor, host RNA silencing and autophagy. The revised still include inappropriate descriptions and errors listed below.

**Part II – Major Issues: Key Experiments Required for Acceptance**
Please use this section to detail the key new experiments or modifications of existing experiments that should be absolutely required to validate study conclusions.

Generally, there should be no more than 3 such required experiments or major modifications for a "Major Revision" recommendation. If more than 3 experiments are necessary to validate the study conclusions, then you are encouraged to recommend "Reject".

Reviewer #1: The use of Arabidopsis ATG12 in Fig. S12. To reveal the role of NbeIF4A in regulation of autophagy, NbATG12 is more appropriate than AtATG12.

Reply: Thanks for the comment. We have now added the experiments to use NaATG12 for analysis. Results were provided in Fig. S12 (Lines 286-295).

Reviewer #2: 1. Fig. S2, no information (nucleotide sequence) of primers for RT-PCR to detect RSV was provided.

Reply: Thanks for the comment. The nucleotide sequence of primers for RT-PCR to detect RSV was now added in the Table S1.

2. L137-141, the authors described "Several defense pathways participate in plant responses to viruses, including RNA silencing, phytohormone signaling, autophagy, and 26S proteasome-mediated degradation. To determine whether these pathways help prevent RSV infection in NbeIF4A-silenced plants, we investigated the expression of marker genes for each pathway upon NbeIF4A silencing." However, The authors did not provide the results, except for those related to autophagy in Fig. S3. Provide the other results or rewrite these sentences.

Reply: Thanks for the comment. We have now rewritten these sentences (Lines 137-140).

3. Fig. 3 and Fig. S6, Describe how the TuMV CP, RSV CP and P3 proteins were detected. If specific antibodies were used, did the authors buy them or prepare by themselves?

Reply: Thanks for the comment. We have now added information on these antibodies in figure legends and in the Materials and Methods (Lines 475-476). These antibodies were prepared in our laboratory.

**Part III – Minor Issues: Editorial and Data Presentation Modifications**
Please use this section for editorial suggestions as well as relatively minor modifications of existing data that would enhance clarity.

Reviewer #1: 1. Page 6, lines 137-144: The text implies that the authors measured the transcript levels of RNAi and other genes related to antiviral defense. However, I could not find the data relevant to this point. Please provide the data or revise the text appropriately.

Reply: Thanks for the comment. We have now revised the text appropriately (Lines 137-140).

2. Page 6, line 162: Provide a reference for the role of NbeIF6A in translation.

Reply: Added (Line 161).

3. Fig. S4: The authors used a 35S-driven GFP construct as a proxy for protein translation. The result supports the authors’ claim but does not rule out that NbeIF4A has any role in RSV translation.
Reply: Agreed. Indeed, it is difficult to design an experiment to exclude such possibility. We have now added a sentence to point it out in the text (Line 169).

4. Fig. 3A-C: The experimental design for this experiment is missing in the main text. The authors might use 16c Nb plants to address the effect of NbeIF4A-silencing on the VSR activity of the RSV p3 protein. It would be helpful if the authors could briefly describe this point in the main text (Page 8).
Reply: Thanks for the comment. We have now added the description on this (Lines 202-205).

5. Page 9, lines 236-238: To be more accurate, the authors should provide all the repeats relevant to Fig. 3F and include statistical analysis of accumulation level of RSV CP in the supplement.

Reply: Thanks for reviewer. we have now added the results in Fig. S8E.

Reviewer #2: L 31, rice stripe virus (RSV) (all lowercase letters and roman)

Reply: Corrected throughout the text.

L 78, "Autophagy-related genes (ATGs) are key factors" should be "Autophagy-related genes (ATGs) encode key factors"

Reply: Corrected (Line 78).

L 104, "encoding eukaryotic translation initiation factor 4A (eIF4A)" should be "encoding eIF4A"

Reply: Corrected (Line 104).

L 182, Is the description: "monitored the progression of infection with the CFP-NbATG8f reporter", OK? with GFP

Reply: Corrected (Line 182).

L 187, "the TuMV-GFP chimeric virus" should be "GFP-tagged TuMV"

Reply: Corrected (Line 187).

Regarding the TuMV-GFP, describe how the authors got the infectious clone and provide the reference regarding the infectious clone of GFP-tagged TuMV.

Reply: We have now added this information by citing a reference (Line 187).

All "Nib" in the text should be "NIb"

Reply: Corrected.

L 196-197, Fig. S6C just investigated the accumulation of NIb-GFP and the authors did not provide evidence proving the degradation of NIb-GFP. Rewrite the sentence.

Reply: Rewritten (Line 195-196).
